# Multiple regions of chromosome 6q affected by loss of heterozygosity in primary human breast carcinomas.

**DOI:** 10.1038/bjc.1996.27

**Published:** 1996-01

**Authors:** Z. M. Sheng, A. Marchetti, F. Buttitta, M. H. Champeme, D. Campani, M. Bistocchi, R. Lidereau, R. Callahan

**Affiliations:** Laboratory of Tumor Immunology and Biology, National Cancer Institute, Bethesda, MD 20892, USA.

## Abstract

**Images:**


					
British Journal of Cancer (1996) 73, 144-147

fft        (C3 1996 Stockton Press All rights reserved 0007-0920/96 $12.00

Multiple regions of chromosome 6q affected by loss of heterozygosity in
primary human breast carcinomas

ZM Sheng', A Marchetti2, F Buttitta2, M-H Champeme3, D Campani2, M Bistocchi2, R Lidereau3
and R Callahan'

Laboratory of Tumor Immunology and Biology, National Cancer Institute, Bethesda, MD 20892; 2Institute of Pathological Anatomy

and Histology, University of Pisa, Pisa, Italy; 3INSERM, Centre Rene Huguenin, 92211 Saint Cloud, France.

Summary A total of 80 primary human breast carcinoma DNAs were analysed for loss of heterozygosity
(LOH) on the long arm of chromosome 6, using microsatellite markers whose location has been defined
physically and by linkage analysis. Loss of heterozygosity was observed in 38 of 80 (48%) tumours that were
informative for at least one locus. The analysis revealed partial or interstitial deletions of chromosome 6q.
Detailed mapping of chromosome 6q in these tumour DNAs identified two and perhaps three commonly
deleted regions. One of these is located between markers D6S251 and D6S252 (6q14 -q16.2), another between
D6S268 and D6S261 (6q 16.3 -q23) and a third between D6S287 and D6S270 (6q22.3 -q23. 1).
Keywords: breast cancer; deletion; chromsome 6q; tumour-suppressor gene

Cytogenetic (Dutrillaux et al., 1990; Lu et al., 1993;
Thompson et al., 1993; Trent et al., 1985, 1993) and
molecular analyses (reviewed in Callahan et al., 1993) of
primary human breast carcinomas have documented fre-
quently occurring genetic alterations that take place during
the evolution of tumour development. It is thought that these
mutations either inactivate normal growth controls or give
the tumour some selective advantage. At the molecular level,
loss of heterozygosity (LOH) is the most frequent type of
genetic alteration in primary human breast tumours
(Callahan et al., 1993). LOH at specific chromosomal loci
has been taken as evidence for the presence of putative
tumour-suppressor genes within the affected regions
(Knudson, 1989). In sporadic primary human breast
carcinomas LOH has been detected on at least 12 different
chromosome arms (Callahan et al., 1993). However only in
the case of chromosome l7pl3 has the target gene for LOH
(TP53) been identified (Hollstein et al., 1991). Generally one
allele of the target gene is lost and the remaining allele
contains a nonsense or missense mutation. The involvement
of chromosome 6q in breast carcinomas has been noted in
cytogenetic analysis of primary tumours (Dutrillaux et al.,
1990; Lu et al., 1993; Thompson et al., 1993; Trent et al.,
1985, 1993). Similarly, molecular analysis of primary breast
tumour DNAs has shown that chromosome 6q is frequently
affected by LOH (Devilee et al., 1991). In this report, we
describe studies aimed at defining the location of putative
tumour-suppressor gene(s) on chromosome 6q in primary
breast tumour DNAs. We have screened 80 pairs of matched
breast tumour and normal DNAs using sequence-tag sites
(STSs) whose location has been defined by linkage analysis
and have constructed a detailed deletion map of this
chromosome arm.

Materials and methods

Thirty invasive ductal carcinomas (IDCs) of the breast and
matching peripheral lymphocytes were collected at the Centre
Rene Huguenin, Saint Cloud, France (tumour panel 1), and
another 50 pairs of IDCs and matched lymphocytes were
collected from University of Pisa, Pisa, Italy (tumour panel

2). Patients corresponding to each of the tumour panels had
received no prior therapy.

Genomic DNA was extracted and diluted to 100-
200 ng pl-'. Polymerase chain reaction (PCR) was per-
formed with 100-200 ng of template DNA, 10 mM Tris-
HCl, 1.5 mM magnesium chloride, 50 mM potassium chloride,
gelatin 0.1 mg ml-', 200 g1M each dNTP, 0.5 U Taq
polymerase (Boehringer Mannheim) and 50 pmol of each
primer in a total volume of 10 1l or 25 Ml. The PCR product

was identified by end labelling primers with [y-32P]ATP or the
PCR product was internally labelled with [c_-32P]dCTP. All

PCR reactions were performed on a Perkin Elmer Cetus PCR
system with denaturation for 6 min at 94?C followed by 30
cycles of denaturation at 94?C for 1 min, annealing
temperature (Table 1) for 1 min, and extension at 72?C for
1 min. The primers for the STS loci that were examined
(Gyapa et al., 1994; Volz et al., 1994), their annealing
temperatures and the references describing them are shown in
Table I.

The PCR products were diluted with loading buffer (95%
formamide, 20 mM EDTA, 0.05% bromophenol blue and
0.05% xylene cyanol), heat denatured and rapidly cooled.
Samples were run in pairs (tumour and lymphocyte PCR
product from the same patient) on a denaturing gel (7%
acrylamide, 32% formamide, 6 M urea, 1 x TBE) at a
constant 30-35 W. After electrophoresis the gel was
transferred to 3MM Whatman paper and autoradiography
performed with Kodak X-Omat AR film at - 70'C. When
the signal of an allele in tumour DNA was less than 50% of
intensity observed in matching normal DNA from a
heterozygous patient, LOH was considered to have occurred
(Bieche et al., 1993).

Results

Preliminary results obtained at 6 loci (D6S254, D6S251,
D6S252, D6S249, ARGI and D6S255) on chromosome 6q in
30 breast tumour DNAs (tumour panel 1) suggested that
LOH on this arm of chromosome 6 was a frequent event
(Table II). LOH was detected in nine tumour DNAs. In three
tumour DNA samples all informative loci were affected by
LOH. Six other tumour DNAs had loss of one allele at
D6S251 or D6S252 or D6S249. However, the data set was
too small to determine with any precision the location of the
target region(s). Therefore, this study was extended to include
another 50 primary breast carcinoma DNAs (tumour panel 2)
with eight additional microsatellite markers whose localisa-

Correspondence: R Callahan, National Cancer Institute, Building 10,
Room 5B50, Bethesda, MD 20892, USA

Received 7 April 1995; revised 11 August 1995; accepted 22 August
1995

LOH on chromosome 6q in breast tumour DNAs
ZM Sheng et al

145
Table I STS loci and primers on chromosome 6q
Annealing
temperature

LocusISTS  (OC)           Primers (5'-3')        References

D6S254    55    AGAGAGGCTGAAGACCAATC         Wilkie et al. (1993)

TCCCATAGCTACAAGCCACT

D6S286    55    GGCCCAGCATCACCCCTAAT         Gyapa et al. (1994)

CCAATCGTGCATCCCAAAGA

D6S284    53    CATGGCTGTCTATCAAACCC         Gyapa et al (1994)

AAGCATTTGTGTGGCTCTTG

D6S251    55    TTCCTAACCAGGTTTCAATG         Wilkie et al (1993)

ATATTTTTAAAGTAAGTTGCVAC

D6S252    55    TGAAAGGAAAGTCCTGCTTC         Wilkie et al. (1993)

ATGGCTCAGGATTCACATTG

D6S249    55    TTCTATTTCTGAAGGTGAACTA       Wilkie et al. (1993)

ATAGTTACCATCAGTCACTG

D6S268    50    CTAGGTGGCAGAGCAACATA         Gyapa et al. (1994)

AAAAGGAGGTCATTTTAATCG

D6S302    50    TTCACAATGACAAGTCCAATACACG    Gyapa et al. 1994)

TTCTTTAGGATAAGCCAATACACG

D6S261    50    GTGAAACCCTGTCTCACTGC         Gyapa et al. (1994)

GGATTTATAGTGACCATGCCA

D6S287    50    ATATTAGTGCCTTATGCTTCTG       Gyapa et al. (1994)

AAATTGGATATTCATGCTTG

D6S262    50    ATTCTTACTGCTGGAAAACCAT       Gyapa et al. (1994)

GGAGCATAGTTACCCTTAAAATC

D6S270    55    GTGTAACTGATCTGAATGGTTCC      Gyapa et al. (1994)

GTAGTGAAGCCTGGATGTGG

ARGI      55    CTACATATTTCTAAATACATGC       Wilkie et al. (1993)

ACTTAGTAGTTTTAAGCAGGA

D6S255    55    TCAGCATCAAGGTACTTGAG         Wilkie et al. (1993)

TTAGTGCCCTATGCAAGGCA

Table II LOH on chromosome 6q in breast tumour panel 1

Regional                         LOH/inf                     Tumour DNA No.

assignment     Locus        n      (%)       14   21    22    25   48    51    54   55    66
6ql3          D6S254        30    0/5 (0)   NI    NI    NI    H    NI    NI   NI    NI    NI
6ql4-q16.2    D6S251        30    6/28 (21)  D    H     D     H     D    D     D     H    D

D6S252        30    4/16 (25)  NI    D    NI    NI    H    D     NI    D     D
6ql6.3-q21    D6S249        30    5/14 (36)  D    NI    H     D     H    D     D    NI    D
6q22.3-q23.1   ARG          30    2/12 (17)  NI   D     NI   NI     H    NI   NI     H    D
6q25.2        D6S255        30    3/9 (33)   D     D    NI   NI    NI    NI    H    NI    D

The genotypes of nine tumour DNA samples from tumour panel 1 at STS markers between D6S254 and
D6S255 that were tested and their regional locations are listed. The genetic order of the STS loci is according to
published linkage studies (Gyapa et al., 1994; Volz et al., 1994). n, total number of tumour DNA samples
examined for each marker; LOH/inf., fraction of tumours from informative patients that showed LOH at each
marker; the number in parenthesis is the percentage of tumours having LOH at each locus; D, LOH; H, STS loci
that were informative but unaffected; NI, STS loci that were not informative.

tion and order were determined by linkage analysis (Gyapa et
al., 1994; Volz et al., 1994).

In this second set of 50 breast tumour DNA samples, all
were informative for at least one locus and of these 29 (58%)
had LOH at one or more loci. The frequency of LOH at the
different STSs loci on chromosome 6q for tumour panel 2 is
shown in Table III. Autoradiographs of two tumour DNA
samples from tumour panel 2, each at three different STS
loci, is shown in Figure 1. Sample 281 had LOH at D6S286
and D6S251 but was informative and unaffected at D6S252.
These results taken together with those summarised in Table
III for tumour DNA samples 127, 28, 91, 304, 263 and 49 are
consistent with the presence of a tumour-suppressor gene
located in the 10 cM interval between D6S284/D6S286 and
D6S252 on chromosome 6ql4-ql6.2 (region 1). If tumour
DNA 82 is also considered the size of region 1 may be
further reduced to the 1.1 cM interval between D6S284/
D6S286 and D6S251.

Shown in Figure 1 are autoradiographs of tumour DNA
sample 82, which indicate that a second region of
chromosome 6q is independently affected by LOH. In this
tumour DNA, D6S268 and D6S261 are both informative and
unaffected whereas LOH was detected at D6S302. The results

summarised in Table III for tumour DNA samples 63, 224
and 204 are also consistent with the presence of a tumour-
suppressor gene located in the 5.2 cM interval between
D6S268 and D6S261 (region 2). Evidence for a third region
affected by LOH was found in tumours DNAs 204, 99, 208,
49, 114 and 83 (Table III). In these tumour DNA samples
D6S262 was affected by LOH, whereas the more centromeric
locus D6S287 was informative and unaffected. The telomeric
boundary of this region could be D6S270 since in tumour
DNAs 204 and 99 it was unaffected by LOH.

Several of the tumour DNAs were remarkable in that
more than one region of chromosome 6q was affected by
LOH. For instance tumour DNA samples 82, 263 and 281
exibited independent LOH of regions 1 and 2 whereas in
tumour DNA 204 regions 2 and 3 were independently
affected by LOH (Table III). Similarly in tumour DNA
sample 208 regions 1 and 3 were affected by LOH and in
sample 49, each of the three regions were independently
affected by LOH. Cytogenetic analysis of metastatic breast
carcinomas have also identified tumours with multiple
chromosome 6 alterations (Dutrillaux et al., 1990; Lu et al.,
1993; Thompson et al., 1993; Trent et al., 1993). In several
cases it was not possible to unambiguously determine which

LOH on chromosome 6q In breast tumour DNAs
$0                                                          ZM Sheng et al

146

(N  ~ ~Z~00

(ZN ~ ~ ~ ~ ~ ~ ~ ~ ~ ~ ~~~(

t n  t)    VI  tn  t >

0      -   (N

'.0      (-4   N     C0%   00    (N                (N

00    0 0     )    m     -    '.          '0    00    '0

.     '.0    .    ' C   ' C   '

go          \0    \0    \0    \0    \0    \0    \

_         (

csN.(            (N
'.         'C    '.

\.0

'IO
'00
-4

00

CNK

00

00,

_

V.0

Q
00

0'.o

00

o(i

(N

o0
00

0

0)

r-

'.0
04

0

0
0s

LOH on dwmorson   6q in brhat bUnu DM

M Shieng et aI                                                     %

147

a                      b

281                    82

T  L I                 T     L

D6S286_     f          D6S268
D6S251                 D6S302
D6S252                 D6S261

Fugwe 1 Autoradiographs of three tumour DNA samples having
LOH at STS markers on chromosome 6q. (a) The STS markers
were D6S286, D6S251 and D6S252. Arrows mark the deleted
allele at D6S286 and D6S251 in tumour DNA 281. (b) The STS
markers were D6S268, D6S302, D6S261. The arrow marks the
deleted aLlele of D6S302 in tumour DNA 82. The autoradiograph
for tumour and lymphocyte DNA at D6S268 represent different
exposures of the same gel.

region was the target for LOH. Thus in tumour DNAs 351
and 86 either region 1 or region 2 could have been the target
for LOH. Similarly, in samples 178, 394, 125, 272, 14, 150,
and 62 either region 2 or region 3 could contain the target
tumour-suppressor gene.

Discussion

Our results confirm the findings of Devilee et al. (1991) that
LOH on chromosome 6q is a frequent event in primary
human breast carcinomas. They detected LOH at MYB and,
or D6S37 in 50% of the tumour DNA samples. The MYB
locus is 5 cM telomeric of D6S270 (Gyapa et al., 1994; Volz
et al., 1994) and D6S37 is at the distal end of chromosome
6q. Our study extends their results by defining three regions

of chromosome 6q that are independently affected by LOH.
During the course of our work Orphanos et al. (1995)
reported two regions on chromosome 6q that are affected by
LOH in human breast tumour DNAs. One of these spans
6ql3-q21 and probably corresponds to regions 1 and 2 in
our data set. The second region in their study was located at
6q21 -q27 and is distal to region 3 in our study. LOH on
chromosome 6q is not unique to breast carcinomas, it has
been detected in 40% of melanomas in the region of 6ql6-
q23 (Millikin et al., 1991). Similarly, Saito et al. (1992) found
that 51 % of ovarian carcinomas had LOH at one or more of
nine loci on chromosome 6q24-q27. In this study the
commonly deleted region was 6q26-q27.

At the present time there are few, if any, candidate target
genes for LOH on chromosome 6q. However, Negrini et al.
(1994) have shown that microcell-mediated transfer of
chromosome 6 into the human breast tumour cell line
MDA-MB-231 inhibits its tumorgenicity in BALB/c-nu, nu
mice as well as causing the cells to age in culture. An analysis
of polymorphic loci which identifies the portions of the
transferred chromosome 6 that were retained in the cell line,
suggested that at least two functional regions of 6q are
important for tumour suppression. One functional region was
at the distal end of 6q near D6S48. Based on current linkage
maps of chromosome 6q (Gyapa et al., 1994; Volz et al.,
1994) the second functional region is located between the
CNR locus (6ql4-ql5) and D6S310 (9 cM telomenrc of
D6S270, see Table III). This second functional region is
consistent with regions 1, 2 and possibly 3 presented in our
study. Clearly the development of a physical map of the
polymorphic STSs loci on chromosome 6q should lead to
further definition of the regions affected by LOH and to the
target gene(s) in primary breast tumours.

Acknwledgemets

This study was supported by the Ligue Nationale de la Lutte
Contre le Cancer (LNCC), the Comites Regionaux des Hauts de
Seine, du Val d'Oise et des Yvelines, Association pour la
Recherche sur le Cancer (ARC) and CNR. ACRO
no. 94.01084.39.

References

BIECHE I, CHAMPEME M-H. MATIFAS F, CROPP CS. CALLAHAN R

AND LIDEREAU R. (1993). Two distinct regions involved in lp
deletion in human primary breast cancer. Cancer Res., 53, 1990-
1994.

CALLAHAN R. CROPP C. MERLO GR, DIELLA F, VENESIO T,

LIDEREAU R AND CAPPA APM. (1993). Genetic and molecular
heterogeneity of breast cancer cells. Clin. Chim. Acta, 217, 63 - 73.
DEVILEE P. VAN VLIET M, VAN SLOUN P, KUIPERS DIJKSHOORN N.

HERMANS J AND PEARSON PL. (1991). Allelotype of human
breast carcinoma: a second major site for loss of heterozygosity is
on chromosome 6q. Oncogene, 6, 1705- 1711.

DUTRILLAUX B. GERBAULT-SEUREAU AND ZAFRANI B. (1990).

Characterization of chromosomal abnormalities in human breast
cancer. Cancer Genet. Cytogenet., 49, 203-217.

FRIEND SH, BERNARDS R, ROGELJ S, WEINBERG RA, RAPAPORT

JM, ALBERT DM AND DRYJA TP. (1986). A human DNA segment
with properties of the gene that predisposes to retinoblastoma and
osteosarcoma. Nature, 323, 643.

GYAPA G, MORISSETTE J, VIGNAL A, DIB C. FIZAMES C. MILL-

ASSEAU P, MARC S, BERNARDI G. LATHROP M AND WEISSEN-
BACH J. (1994). The 1993-94 Genethon human genetic map.
Nature Genet., 7, 246- 339.

HOLLSTEIN M, SIDRANSKY D, VOGELSTEIN B AND HARRIS CC.

(1991). p53 mutations in human cancers. Science, 253, 49- 53.

KNUDSON AG. (1989). Hereditary cancers: clue to mechanisms of

carcinogenesis. Br. J. Cancer, 59, 661-666.

LU Y-J. XIAO S. YAN Y-S. FU S-B. LIU Q-Z AND LI P. (1993). Direct

chromosome analysis of 50 primary breast carcinomas. Cancer
Genet. Cvtogenet., 69, 91-99.

MILLIKIN D, MEESE E. VOGELSTEIN B, WITKOWSKI C AND TRENT

JM. (1991). Loss of heterozygosity for loci on the long arm of
chromosome 6 in human malignant melanoma. Cancer Res., 51,
5433-5449.

NEGRINI M. SABBIONI S. POSSATI L. RATTAN S. CORALLINI A.

BARBANTI-BRODANO G AND CROCE CM. (1994). Suppression
of tumorigenicity of breast cancer cells by microcell-mediated
chromosome transfer: Studies on chromosomes 6 and 11. Cancer
Res., 54, 1331-1336.

ORPHANOS V, MCGOWN G. HEY Y, BOYLE JM AND SANTIBANEZ-

KOREF M. (1995). Proximal 6q, a region showing allele loss in
primary human breast cancer. Br. J. Cancer, 71, 290 -293.

SAITO S, SAITO H, KOOI S. SAGAE S. KUDO R, SAITO J. NODA K

AND NAKAMURA Y. (1992). Fine-scale deletion mapping of the
distal long arm of chromosome 6 in 70 human ovarian cancers.
Cancer Res., 52, 5815- 5817.

THOMPSON F, EMERSON 1, DALTO J-M, MCGEE D. VILLAR H.

KNOX S, MASSEY K, WEINSTEIN R, BHATTACHARYYA A AND
TRENT J. (1993). Clonal chromosome abnormalities in human
breast carcinomas I. Twenty-eight cases with primary disease.
Genes, Chrom. Cancer, 7, 185-193.

TRENTJM. (1985). Cytogenetic and molecular biologic alterations in

human breast cancer: a review. Breast Cancer Res. Treat., 5, 221-
229.

TRENT J, JIN-MING Y. EMERSON J. DALTON W, MCGEE D. MASSEY

K, THOMPSON F AND VILLAR H. (1993). Clonal chromosome
abnormalities in human breast carcinomas II. Thirty-four cases
with metastatic disease. Genes, Chrom. Cancer, 7, 194- 203.

VOLZ A. BOYLE JM. CANN HM. COTTINGHAM RW. ORR HT AND

ZIEGLER A. (1994). Report of the second workshop on human
chromosome 6. Genomics. 21, 464-472.

WILKIE P. POLYMEROPOULOS MH. TRENT JM, SMALL KW AND

WEBER JL. (1993). Genetic and physical map of 11 short tandem
repeat polymorphisms on human chromosome 6. Genomics, 15,
225-227.

				


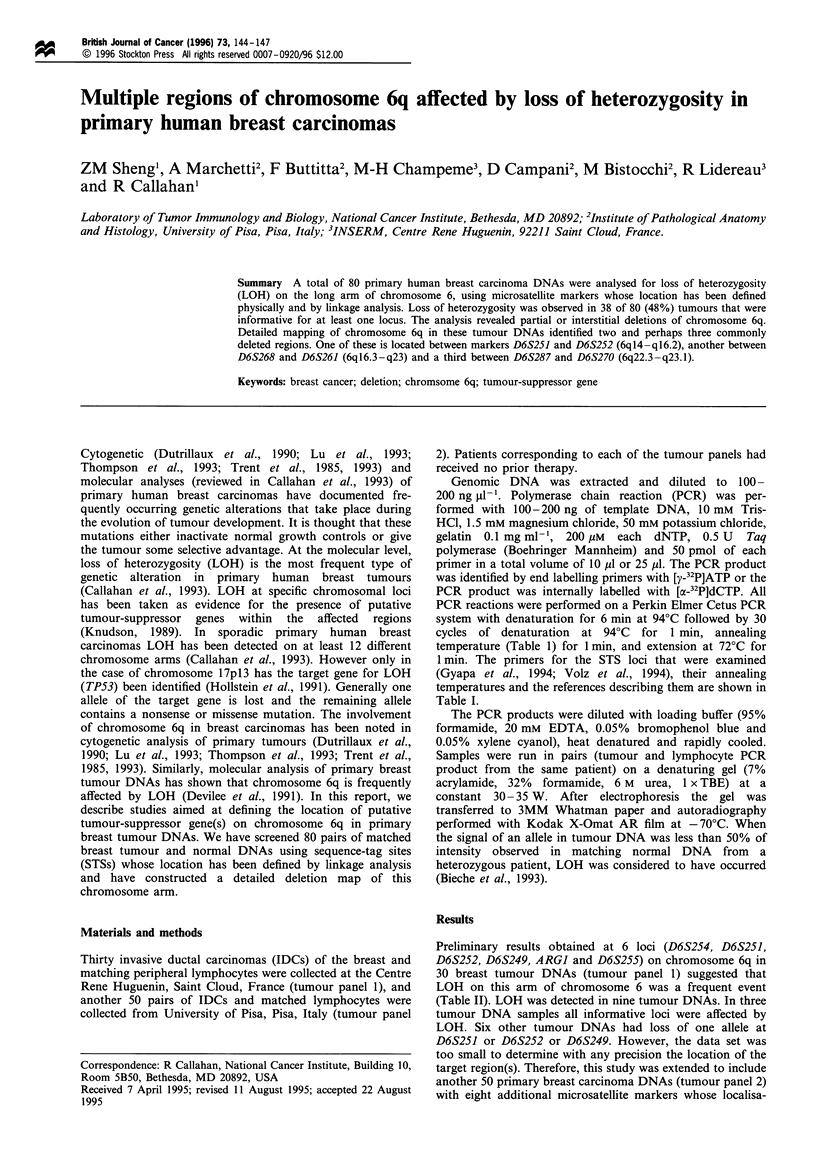

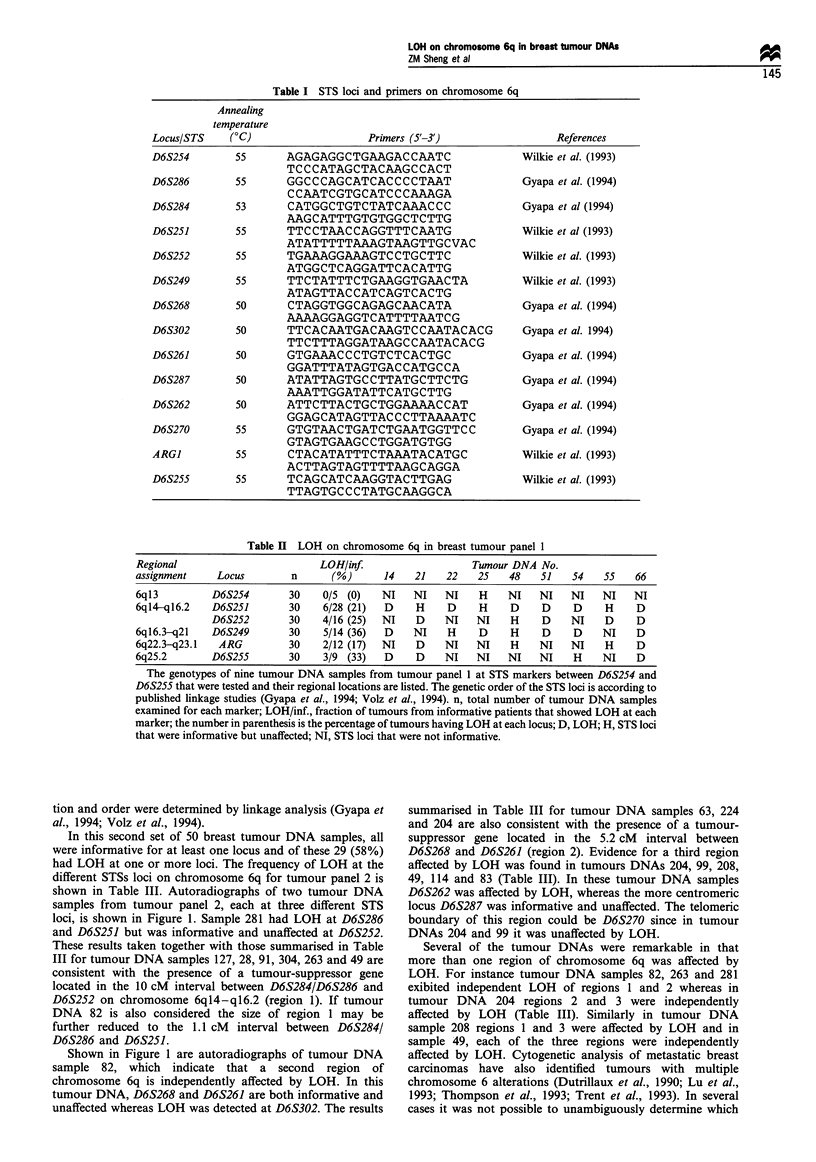

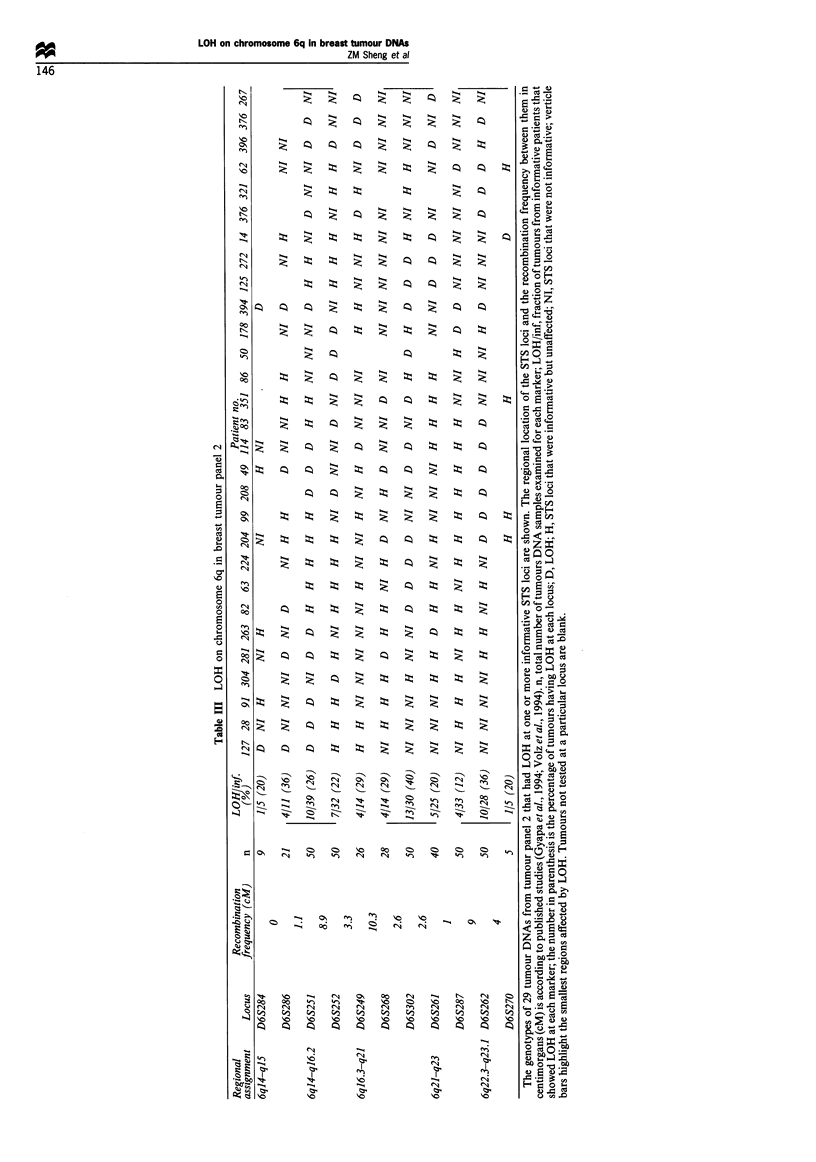

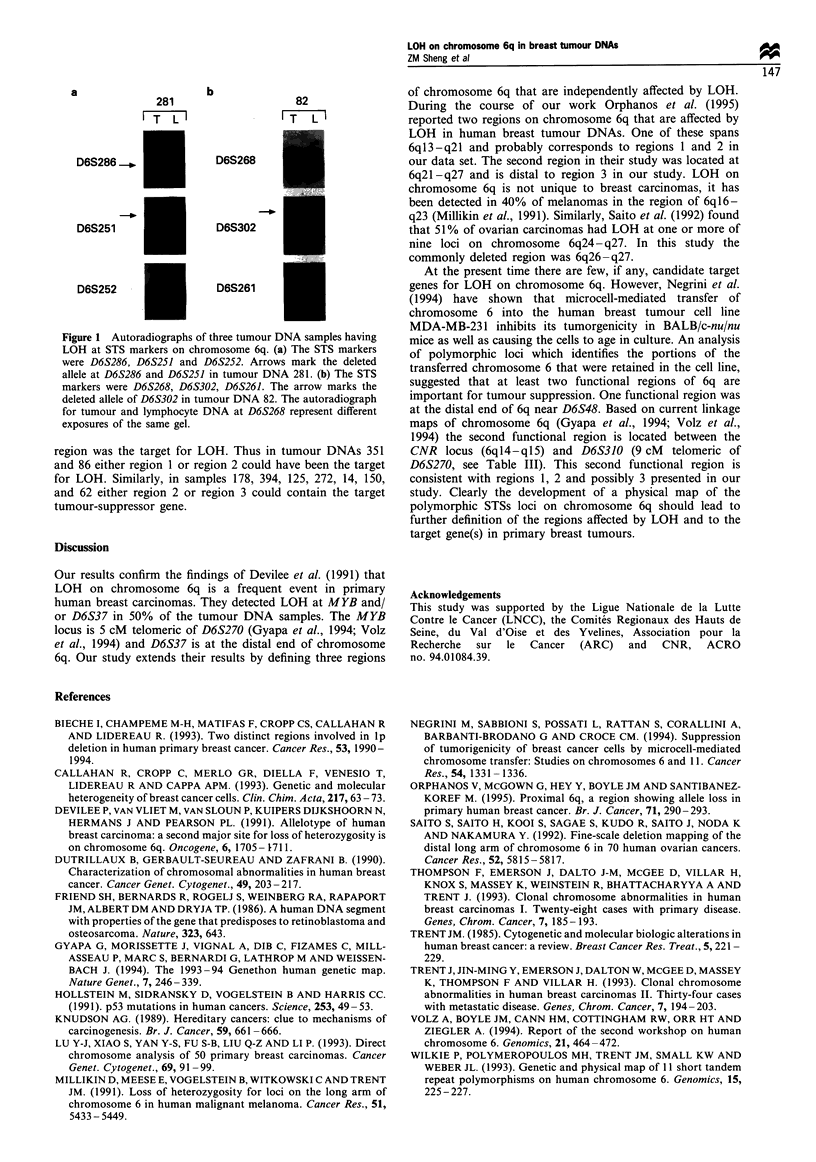

